# The Use of Liquid Rubber in Dentistry

**Published:** 1907-08-15

**Authors:** J. T. Nelson


### THE USE OF LIQUID RUBBER IN DENTISTRY.

BY J. T. NELSON, D.D.S.

I believe the subject of liquid rubber is one that deserves our most careful investigation. I believe it is a future necessity and a present possibility that in the past has been sadly neglected. By liquid rubber we usually mean vulcanite rubber cut with chloroform and used for veneering and other like work on the model or plate out of the mouth. While this rubber is used to some extent, it does not, in the majority of cases, shorten the time nor make the work better, and we prefer to use common vulcanite rubber in the same old way.

Until recently this has been the only form of liquid rubber available. But there is now on the market a preparation known as the Nelson-Reid Liquid Dental Rubber for Refitting and Mending Rubber Plates. It is of this and its use that I wish to speak for a few moments.

I believe the profession has been waiting a good many years for a liquid rubber that can be used in the mouth as well as out, and that is a filler as well as a lining or veneer. The Nelson-Reid rubber has these qualities. It is put up in the form of a paste mixed to such a consistency that it will not run and at the same time soft enough to take an accurate impression. It can be had in the three colors—red, black and pink.

I have been using it in my practice for the past six months, during which time I have tested it thoroughly from all points of view. For refitting I find it has no superior. Now, it stands to reason, that any substance of the consistency of paste will spread and the surplus squeeze out as long as we exert pressure. Thus we can, if we desire, squeeze out all except a very thin layer. The process of taking the impression is much the same as for plastic rubber, except that the liquid rubber requires no heat. The palatal surface of the loose plate is cleaned and dried and a thin layer of liquid rubber spread over it. Then the plate is placed in the mouth and the impression taken in the liquid rubber. Then the plate is removed and laid aside for forty-five minutes to allow the rubber to become hard. At the end of this time the impression is renewed, after which it is flasked and vulcanized.

There is nothing so beneficial to the profession as an article or device for saving time and work. In the use of liquid rubber for refitting we have shortened the time a little and obtained better results. Now the mending proposition, I believe, will interest us even more than the refitting. The broken edges are cleaned with chloroform and a little of the liquid rubber spread on them. Then they are held together firmly for two or three minutes in the position desired when finished. This operation is as simple as waxing it for the old-fashioned mend. Now it is ready to flask and vulcanize. Did we save any time? There was no waxing together; no opening the flask; no cutting out of old rubber and packing of new.

We say, "This is all very well, but will it work?" I want to say to you, "It will work." It will come nearer giving a perfect and invisible mend than any process I know. The rubber, being liquid, runs into the tiny pores and crevices and thus reaches out quite a distance into the old rubber. In all my tests of plates that have been mended with liquid rubber, I find that the plate almost invariably breaks in a new place. This shows that the mend is a strong one and that is the all important point.

There are four questions that will come to your minds and I want to answer them before we go further.

No. 1. Will this rubber affect the soft tissues while taking the impression?

No, it will not. There is no unpleasant taste nor smarting effect, and the taking of modeling compound impression disturbs the soft tissues more than does liquid rubber. A little examination will show that the drugs used in cutting the rubber are almost entirely non-irritant.

No. 2. Will it stick to the mouth?

Modeling compound, when heated, will stick to the dry fingers, if you wet them it will not stick, or if you place it in the mouth it will not stick. While liquid rubber sticks tenaciously to the plate or any dry surface, the moisture in the mouth prevents its sticking there. If the directions were followed, there will be no sticking to the mouth.

No. 3. Will it vulcanize hard enough? In what time? At what temperature?

When we understand the principle of the working of this rubber, the question of its vulcanization is no longer a question. The rubber is put in the liquid form in order that we may take an impression in it or stick our broken plate together. After this is accomplished, we are done with the liquid form, in fact, our work cannot go on as long as it is liquid. In this form it would never vulcanize. The drugs used for cutting the rubber entirely disappear by evaporation. Now, we have common dental rubber instead of liquid rubber. This we know will vulcanize in fifty-five minutes at 320 degrees, and as hard as any rubber.

No. 4. Will it shrink?

My answer to this is, yes. In the evaporation of the liquids there is a little shrinkage. But after we have waited forty-five minutes and renewed the impression this shrinkage is taken up, and if we flask immediately, there is no chance for more shrinkage.

In mending, the shrinkage only draws the edges closer together and is an advantage as it gives a closer joint.

Now, just a word in behalf of refitting. There are men who tear down and tear down good, bad, and indifferent plates. I have some plates that I should be glad to tear down and make over and others that are better than I could get them again. I have also seen plates made by other dentists that I knew were far better than I could make them, but they were loose and something had to be done. When a patient has worn a plate for some time, he usually wants it left as it is or, when made over, to be just the same. The latter is an impossible task and a man is foolish to undertake it. In these cases I say refit. You can do it a little cheaper, and, if your time is worth much, make a little more money than by tearing down and building over. Your patient will be better pleased, and you will be better pleased; your patient's bank account will not decrease so much as he expected, and yours will increase a little more than you expected.—*Western Dental Journal.*

